# Supplementary material for: Preventable Disease, the Case of Colorado: School District Demographics and Childhood Immunizations
Source: Vaccines (Basel). 2022 Sep 21;10(10):1579. doi: 10.3390/vaccines10101579 (PMC9607491; doi:10.3390/vaccines10101579)
Supplement: Supplementary file 1 [file vaccines-10-01579-s001.zip › vaccines-1893195-supplementary.pdf]

Supplementary Table S1. Colorado school district compliance and exemption type rates by vaccine. Mean, SD, Minimum and Maximum values estimated from School District aggregate data.

	Parameter	Fully Immunized %	In Process %	Incomplete Record %	Medical Exemption %	No Record %	Personal Exemption %	Religious Exemption %	Compliant For All %
**All**	**Mean**	92.7%	0.6%	1.8%	0.1%	0.8%	3.7%	0.3%	94.7%
	**Std Dev**	6.7%	2.1%	3.4%	0.4%	2.3%	4.1%	0.7%	7.8%
	**Minimum**	28.3%	0.0%	0.0%	0.0%	0.0%	0.0%	0.0%	39.3%
	**Maximum**	100.0%	33.6%	51.5%	15.1%	70.7%	48.8%	8.3%	100.0%
**DTaP**	**Mean**	93.8%	0.4%	1.4%	0.1%	0.7%	3.4%	0.3%	94.8%
	**Std Dev**	5.7%	1.3%	2.8%	0.3%	1.6%	3.7%	0.7%	7.8%
	**Minimum**	44.7%	0.0%	0.0%	0.0%	0.0%	0.0%	0.0%	39.3%
	**Maximum**	100.0%	21.7%	40.9%	6.8%	16.3%	40.6%	7.7%	100.0%
**HepB**	**Mean**	94.0%	0.4%	1.1%	0.1%	0.7%	3.5%	0.3%	94.8%
	**Std Dev**	5.6%	1.4%	2.0%	0.3%	1.7%	3.9%	0.7%	7.8%
	**Minimum**	44.2%	0.0%	0.0%	0.0%	0.0%	0.0%	0.0%	39.3%
	**Maximum**	100.0%	22.1%	22.5%	6.8%	16.3%	39.7%	7.7%	100.0%
**MMR**	**Mean**	93.5%	0.4%	1.3%	0.1%	0.7%	3.6%	0.3%	94.8%
	**Std Dev**	5.8%	1.4%	2.2%	0.3%	1.8%	4.0%	0.7%	7.8%
	**Minimum**	45.2%	0.0%	0.0%	0.0%	0.0%	0.0%	0.0%	39.3%
	**Maximum**	100.0%	21.2%	23.8%	6.8%	18.7%	42.0%	7.2%	100.0%
**Polio**	**Mean**	93.3%	0.5%	1.5%	0.1%	0.7%	3.7%	0.3%	94.8%
	**Std Dev**	5.9%	1.4%	2.5%	0.3%	1.7%	4.0%	0.7%	7.8%
	**Minimum**	43.8%	0.0%	0.0%	0.0%	0.0%	0.0%	0.0%	39.3%
	**Maximum**	100.0%	22.6%	23.8%	6.8%	16.3%	41.1%	7.7%	100.0%
**Tdap**	**Mean**	89.6%	1.3%	3.6%	0.1%	1.2%	3.9%	0.3%	94.3%
	**Std Dev**	9.2%	3.8%	5.8%	0.6%	4.1%	4.8%	0.8%	8.3%
	**Minimum**	28.3%	0.0%	0.0%	0.0%	0.0%	0.0%	0.0%	39.3%
	**Maximum**	100.0%	33.6%	51.5%	15.1%	70.7%	48.8%	8.3%	100.0%
**Varicella**	**Mean**	92.2%	0.6%	1.8%	0.2%	0.8%	4.1%	0.3%	94.8%
	**Std Dev**	6.5%	1.7%	3.1%	0.5%	1.8%	4.4%	0.7%	7.8%
	**Minimum**	42.4%	0.0%	0.0%	0.0%	0.0%	0.0%	0.0%	39.3%
	**Maximum**	100.0%	24.0%	35.0%	6.8%	16.3%	44.3%	7.2%	100.0%
